# AI-driven dual-task prediction model for co-stratifying efficacy and toxicity in NSCLC immunotherapy

**DOI:** 10.1371/journal.pdig.0001568

**Published:** 2026-08-13

**Authors:** Hanlin Ding, Yuting Ren, Siqi Ding, Yuzhong Chen, Yuemin Wu, Yipeng Feng, Wenjie Xia, Xuming Song, Rutao Li, Qixing Mao, Bing Chen, Hui Wang, Bin Zhu, Anpeng Wang, Lin Xu, Yan Qiang, Gaochao Dong, Feng Jiang

**Affiliations:** 1 Department of Thoracic Surgery, Nanjing Medical University Affiliated Cancer Hospital & Jiangsu Cancer Hospital & Jiangsu Institute of Cancer Research, Nanjing, China; 2 Jiangsu Key Laboratory of Innovative Cancer Diagnosis & Therapeutics, Cancer Institute of Jiangsu Province, Nanjing, China; 3 The Fourth Clinical College of Nanjing Medical University, Nanjing, China; 4 The School of Medical Imaging, Nanjing Medical University, Nanjing, China; 5 Department of Oncology, the First Affiliated Hospital with Nanjing Medical University, Nanjing, China; 6 Department of Thoracic Surgery, Dushu Lake Hospital Affiliated to Soochow University, Suzhou, China; 7 Hospital Development Management Office, Nanjing Medical University, Nanjing, China; 8 Department of Oncology, the First Affiliated Hospital with Nanjing Medical University, Nanjing, Jiangsu, China; 9 Collaborative Innovation Center for Cancer Personalized Medicine, Nanjing Medical University, Nanjing, China; 10 Department of Intensive Care Unit, Nanjing Medical University Affiliated Cancer Hospital & Jiangsu Institute of Cancer Research, Nanjing, China; Medtronic and West Virginia University, UNITED STATES OF AMERICA

## Abstract

While effective against non-small cell lung cancer (NSCLC), PD-1 inhibitors can induce immune-related adverse events (irAEs), occurring in up to 15.2% of patients and potentially fatal. Currently, effective predictive biomarkers capable of simultaneously forecasting both irAEs and immune checkpoint inhibitor (ICI) responders remain elusive. This limitation hinders the safe clinical application of these agents. This study enrolled 333 advanced NSCLC patients treated with PD-1 inhibitor monotherapy or combination therapy. CT imaging features were extracted using radiomics and deep-learning approaches. Three unimodal and two multimodal models were constructed to predict irAEs (Grade ≥3) and ICI responders in parallel. The SHAP algorithm was used to identify clinical features contributing to the prediction of both irAEs and ICI responders. The CDML–DenseNet model, integrating clinical features with deep-learning–derived radiomics features (DenseNet), demonstrated superior performance in predicting irAEs (AUC = 0.85), outperforming single-modal radiomics models. For ICI responder prediction, the CDML–DenseNet model achieved an AUC of 0.866. The Prognostic Nutritional Index (PNI) was identified as a key feature in both irAEs and ICI responder prediction models. Patients who were non-responders to ICIs but experienced irAEs had significantly lower PNI (46.8 ± 8.779, P < 0.05) compared with ICI responders without irAEs. Our multimodal CDML–DenseNet model effectively predicts both irAEs and ICI responders in NSCLC patients receiving PD-1 inhibitors. This approach provides a novel framework for balancing immunotherapy efficacy and toxicity. Furthermore, the readily available and cost-effective PNI offers clinicians a practical tool to identify potential non-responders experiencing irAEs and to refine treatment decisions.

## Author summary

We developed a computer-based tool to help doctors predict both the benefits and risks of immunotherapy for patients with advanced lung cancer. While immune therapy has transformed cancer care and improved survival, it can trigger severe inflammation and organ damage in some patients. Currently, physicians lack reliable methods to identify who will respond well and who might suffer harmful side effects before starting treatment, which makes balancing benefit and harm challenging in daily practice.

By combining routine clinical information with imaging analysis through artificial intelligence, we built a prediction system that forecasts treatment success and side-effect risk in parallel. We discovered that a simple nutritional indicator, calculated from standard blood tests measuring albumin and lymphocyte levels, serves as a critical warning sign. Patients with poor nutritional status were more likely to experience serious complications without gaining therapeutic benefits.

Our work offers a practical strategy to personalize cancer treatment, helping physicians weigh potential benefits against risks for each individual. By flagging high-risk patients early, this approach could improve patient safety and support more informed, balanced treatment decisions.

## 1 Introduction

Nowadays, the immune checkpoint inhibitors (ICIs) have improved long-term survival in patients with advanced non-small cell lung cancer (NSCLC) by activating anti-tumor immunity and are recommended as standard first-line therapy for this disease [1,2]. However, despite their clinical benefits, immune-related adverse events (irAEs), a distinct group of organ-specific inflammatory toxicities, pose significant challenges in clinical management [3]. Previous clinical research has shown that most patients receiving ICI therapy experience varying degrees of irAEs. Among these patients, approximately 10% develop Grade ≥3 irAEs, which can affect multiple organs, lead to treatment discontinuation, and may be life-threatening [4–7]. Checkpoint inhibitor pneumonitis (CIP) is the most common fatal irAE, accounting for 35% of all irAE-related deaths [8–10]. Although immune-related myocarditis is uncommon, its fatality rate is as high as 50% [11]. Therefore, monitoring and timely intervention for irAEs during clinical follow-up are crucial.

Although irAEs markedly affect patient prognosis, highly sensitive and specific predictive biomarkers for their monitoring remain lacking. Currently reported peripheral immune indicators, such as the neutrophil-to-lymphocyte ratio (NLR), C-reactive protein, and systemic immune-inflammation index (SII), show limited discriminatory capacity (AUCs ranging from 0.506 to 0.612 for irAE prediction) [8,12]. Although genomic markers, including TMB, MSI status, and transcriptomic signatures, hold promise, their high detection costs (e.g., NGS-based TMB testing at ~€1538 and €261 for conventional PCR) and technical complexity hinder widespread clinical translation, leading to underutilization and missed therapeutic opportunities [13–16]. On the other hand, the complex relationship between ICI responder and irAEs remains unclear [17]. Previous studies have largely focused on either treatment response [18] or irAE occurrence [19], which may not adequately address real-world clinical needs for benefit-risk optimization [20]. As reported by Heyward et al., in patients at high risk of irAEs, prioritizing survival benefits may justify combining chemotherapy with ICIs to prolong overall survival [21]. Conversely, ICIs should be used with extreme caution in individuals unlikely to benefit and at a high risk of severe irAEs. Therefore, integrated evaluation of both ICI response and irAE risk for each patient could enable more precise and effective treatment strategies. In recent years, the application of artificial intelligence (AI) in medical imaging has created new opportunities for lung cancer diagnosis and treatment. Prior studies have demonstrated the feasibility of radiomics or deep-learning models for predicting immunotherapy outcomes [22–24]. Vanguri et al. reported that multimodal fusion models outperform single-modality models in predicting ICI response in NSCLC [25]. However, few studies have addressed irAE prediction. Existing work is often limited by small sample size or single-modality approaches and does not fully exploit the rich information contained in CT images [19,26]. Moreover, no universally accepted high-performance model currently exists for jointly predicting ICI response and toxicity.

**Fig 1:**
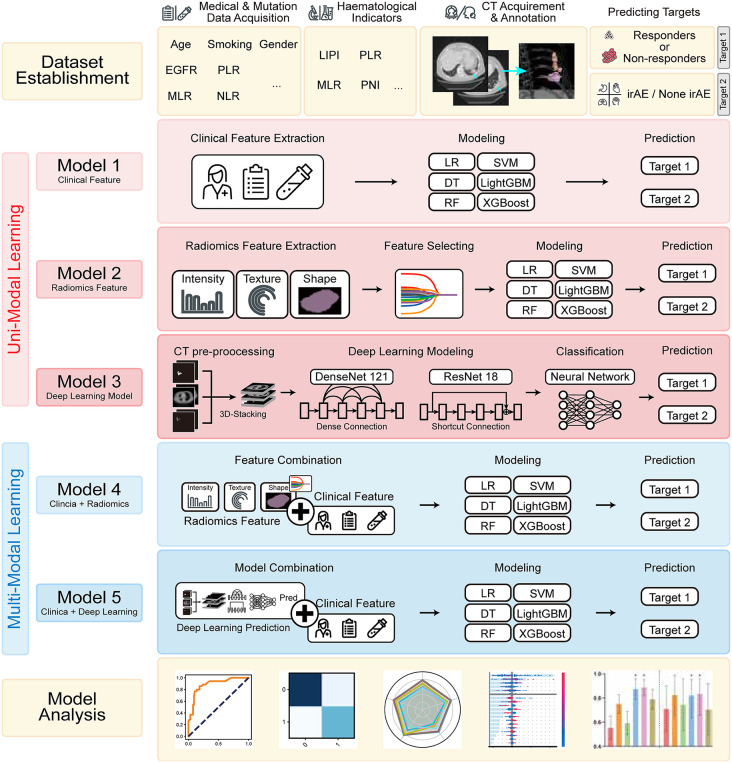
An overview of the framework of unimodal and multimodal models for iRAEs/ICI responder prediction. **Top panel**: Data acquisition and preprocessing, including clinical parameters, hematological indices, and CT-imaging acquisition. **Middle panel**: Five predictive models categorized into Unimodal Learning Models 1–3 and Multimodal Learning Models 4–5. **Bottom section**: Model evaluation and analysis using ROC curves, confusion matrices, radar charts, SHAP analysis, and comparative bar plots. EGFR, Epidermal Growth Factor Receptor; PLR, Platelet-to-Lymphocyte Ratio; MLR, Monocyte-to-Lymphocyte Ratio; NLR, Neutrophil-to-Lymphocyte Ratio; LIPI, Lung Immune Prognostic Index; PNI, Prognostic Nutritional Index; irAE, immune-related Adverse Events; LR, Logistic Regression; SVM, Support Vector Machine; DT, Decision Tree; LightGBM, Light Gradient Boosting Machine; RF, Random Forest; XGBoost, eXtreme Gradient Boosting.

In summary, there is an urgent need to develop a multimodal model, integrating imaging, clinical parameters, and blood biomarkers to simultaneously predict irAEs and ICI response at the individual level. Specifically, this study was conducted in four stages (**Fig 1**): first, clinical data and CT images were collected from patients with advanced NSCLC to establish a well-annotated prospective immunotherapy cohort. Second, radiomic features were extracted from CT images, and deep features were generated using a deep-learning network. Third, three unimodal models (Models 1–3: Clinical feature-based machine-learning [CML] model, the Radiomics-based machine-learning [RML] model, and CT-based deep-learning [CTDL] model) and two multimodal fusion models (Models 4–5: Clinical–Radiomics machine-learning [CRML] model, and Clinical–deep-learning machine-learning [CDML] model) were constructed. Finally, model performance was evaluated and compared using ROC curves, AUC values, and SHAP analysis, and the key clinical features contributing to model predictions were identified as reliable predictive indicators.

## 2 Methods and materials

### 2.1 Ethics statement

This study was approved by the Ethics Committee of Jiangsu Cancer Hospital (No. 2022–035) and followed good clinical practice guidelines in accordance with the principles outlined in the Declaration of Helsinki. Considering the retrospective nature of the study, informed consent was waived.

### 2.2 Study population

Patients with advanced NSCLC who received PD-1 inhibitor therapy between June 2018 and June 2022 at Jiangsu Cancer Hospital were recruited for this study, with or without chemotherapy or anti-angiogenesis therapy. The PD-1 inhibitors used included pembrolizumab, nivolumab, sintilimab, and camrelizumab. Chemotherapy regimens consisted of platinum combined with agents, such as pemetrexed, docetaxel, gemcitabine, or paclitaxel/nab-paclitaxel, while bevacizumab was used as the anti-angiogenesis drug. Inclusion criteria were: 1) age ≥ 18 years; 2) histologically confirmed locally advanced or metastatic NSCLC; (stage IIIB–IV); treatment line ≤3; and a follow-up period of at least 1 year. Exclusion criteria included receipt of radiotherapy during immunotherapy; prior treatment with PD-L1 inhibitors; a history of autoimmune disease; absence of high-quality baseline chest contrast-enhanced CT images (within 30 days before ICI treatment); and loss to follow-up or incomplete data. Data collection ended on 31 January 2024.

Tumors were assessed using CT and cranial magnetic resonance imaging (for brain metastases) approximately every 6 weeks after treatment initiation, and efficacy was evaluated according to Response Evaluation Criteria in Solid Tumors (RECIST version 1.1). Responders were defined as patients whose best overall response was complete response (CR), partial response (PR), or durable stable disease (SD), consistent with the criteria established by Luo et al.[27].

Collected data included demographic characteristics (age, sex), clinical features (smoking history, family history), tumor characteristics (stage, histological type, degree of differentiation, metastasis), treatment information (regimen, line of therapy, driver gene mutation status, PD-L1 tumor proportion score [TPS]), and routine blood test indicators. The Prognostic Nutritional Index (PNI) was calculated as: “PNI = serum albumin (g/L) + 5 × absolute lymphocyte count (×10⁹/L).” The dataset comprised baseline clinical and CT imaging data before treatment, as well as follow-up data until disease progression or death, and clinical and imaging information from the last follow-up, ensuring comprehensive documentation of the treatment course and adverse events.

To validate the generalizability of shared clinical features that jointly predict both ICI responsiveness and irAEs incidence, a previously reported cohort from Jiangsu Provincial Hospital was used as an external validation set, including corresponding clinical data [19].

### 2.3 Evaluation of irAEs

Patients’ irAEs were defined according to the ESMO Clinical Practice Guideline [28], based on (in hierarchical order) pathological confirmation, multidisciplinary committee judgment, or clinical improvement following irAE-directed treatment, after excluding alternative causes. irAEs were monitored from treatment initiation until 1 year after cessation to capture delayed events [9]. IrAEs are graded from 1 to 5: 1–2 indicate mild to moderate, grades 3–4 severe to life-threatening events, and grade 5 is death. In this study, Grade ≥3 irAEs were classified as “irAEs.” To ensure data quality, two physicians independently reviewed the medical records; discrepancies were resolved through discussion with a third senior physician to reach consensus.

### 2.4 Image acquisition and preprocessing

CT data were obtained from the radiology department of Jiangsu Cancer Hospital using LightSpeed VCT equipment. The scanning matrix was set to 512 × 512 pixels, with a slice thickness of 0.625 mm and a reconstructed thickness of 1.25 mm. Lesions were independently annotated by two experienced radiologists using Pair v2.9 software. In cases of disagreement, consensus was reached through consultation with a senior radiologist. CT images in DICOM format were converted into 3D compressed images (nii.gz) using the SimpleITK package for subsequent analysis [29]. Corresponding lesion annotation files (JSON format) and reconstructed 3D images (NIfTI format) were exported via Pair v2.9.

Radiomic feature extraction was performed using the open-source PyRadiomics package, yielding 107 features: 18 first-order features, 14 shape features, and 75 second-order features, including 24 gray-level co-occurrence matrix (GLCM), 16 gray-level size zone matrix (GLSZM), 16 gray-level run-length matrix (GLRLM), 5 neighboring gray-level difference matrix (NGTDM), and 14 gray-level dependence matrix (GLDM) features (**S1 Table**).

For deep-learning model construction, all 3D CT images were resampled to an isotropic resolution of 1 × 1 × 1 mm^3^ using trilinear interpolation. Based on radiologist-annotated lesion regions, ROIs were segmented and reconstructed into lung window (window width 1400 HU, window level −500 HU) and mediastinal window (window width 350 HU, window level 40 HU) image sequences. The original images and lung/ mediastinal window images were stacked to form a 4D CT tensor (height × width × depth × channels) as input to the deep-learning models. The workflow is shown in **S1 Fig**.

### 2.5 Construction of uni-/ multimodal prediction models

#### 2.5.1 Data preprocessing.

In this study, we predicted two outcomes based on the same models: whether a patient is an ICI responder and whether a patient experiences irAEs of Grade ≥3. We labeled the non-responders and patients without Grade ≥3 irAEs as 0, whereas others were labeled as 1. During the training, for predicting irAEs, considering the significant disparity in the number of patients between the two categories, we applied Synthetic Minority Over-sampling Technique (SMOTE) to address the bias induced by data imbalance [30]. This effect was implemented using the imblearn package to balance the dataset before modeling. However, such balancing was not required for modeling ICI responders. The dataset was then split into a training and test set through stratified random sampling at a 8:2 ratio, ensuring that the proportion of samples in each category in the two datasets was consistent with the original dataset.

#### 2.5.2 Model construction.

In this study, three unimodal models were initially constructed, along with two multimodal models. For the machine-learning models, 12 algorithms were evaluated, including k-Nearest Neighbors, Decision Trees, Support Vector Machines (SVC-linear/poly/rbf/sigmoid), NuSVC, LinearSVC, Random Forests (RF), LightGBM, Logistic Regression (LR), and XGBoost.

**Model 1: Clinical feature-based unimodal machine-learning model (CML)**

The clinical model integrated patients’ demographic characteristics, clinical features, and laboratory indicators. All features were standardized using Z-scores and input into the 12 machine-learning algorithms. For both ICI response and irAE prediction, the algorithm with the highest ROC AUC on the test set was selected as the final CML model.

**Model 2: Radiomics-based unimodal machine-learning model (RML)**

Radiomic features were Z-score normalized, and those with an intraclass correlation coefficient (ICC) >0.7 were retained. The Least Absolute Shrinkage and Selection Operator (LASSO) with 5-fold cross-validation was then applied for feature selection. The optimal penalty parameter (α) was determined by minimizing the mean squared error (MSE) and features with non-zero coefficients were selected using the SelectFromModel method. A coefficient path plot against log(α) was used to assess feature stability. The selected features were subsequently input into 12 machine-learning algorithms, and the best-performing model for each task was chosen as the RML model based on ROC AUC.

**Model 3: CT-based unimodal deep-learning model (CTDL)**

Utilizing the preprocessed 4D-stacked CT images, deep-learning models were developed based on ResNet-18 and DenseNet-121.

During training, input data were propagated forward through the network, the loss function was computed, and model parameters were updated via back propagation. Cross-entropy loss and the Adam optimizer were used using the following formula:


$$\begin{array}{c} \text{H}(\text{X})=-\sum_{\text{i}=\text{1}}^{\text{n}}\text{P}\left({\text{x}}_{\text{i}}\right)\text{lo}\text{g}\left(\text{P}\left({\text{x}}_{\text{i}}\right)\right) \end{array}$$ (1)

Where, xi represents a discrete variable, signifying the predicted value for each category of the results in this study.

Upon completion of model training, the AUC was evaluated to select the better performed model as the CTDL model. Moreover, the prediction metrics were converted into a range of 0–1, termed as the deep-learning score (DL_score). Serving as the final output indicator, the DL_score quantifies the model’s prediction confidence for the input data and helps in multimodal fusion analysis.

**Model 4: Clinical–radiomics based multimodal machine-learning model (CRML)**

To further enhance the predictive performance for the two tasks, we developed a multimodal prediction model integrating different data. Initially, 12 machine-learning algorithms based on clinical features and LASSO-selected radiomics features were constructed. Based on the assessment of the ROC AUC values, the optimal multimodal prediction model of the two tasks were selected as the CRML models.

**Model 5: Clinical–DL-score-based multimodal machine-learning model (CDML)**

Finally, we constructed another multimodal prediction model incorporating clinical features and the prediction results from the DL models. A total of 12 machine-learning algorithms were used for modeling, and the best model was selected with the assessment of the ROC AUC value, termed as CDML.

#### 2.5.3 Software and hardware.

The data processing, feature extraction, and model construction were conducted using Python 3.9. For radiomics feature extraction, the PyRadiomics (Version 3.1.0) package was used [31]. Machine-learning model construction and evaluation were performed using Scikit-learn package (1.3.1) [32]. The deep-learning models were implemented based on the PyTorch (2.1.0) and MONAI (1.4.0) frameworks [33]. In addition, data processing and visualization were conducted using Pandas (2.1.1), NumPy (1.26.0), Matplotlib(3.8.0), Seaborn (0.13.0), SimpleITK (2.3.1), SHAP (0.44.0), and PyCM (4.0) [34].

The hardware environment consisted of two EPYC 7601 CPUs, 512GB of RAM and two Nvidia RTX 3090 (24GB) GPUs. The training of deep-learning models was performed on the NVIDIA GPU, whereas feature extraction and machine-learning model training were executed on the CPU.

### 2.6 Statistical analysis

Baseline characteristics were described as follows: continuous variables were expressed as medians (interquartile range) and compared between the groups using the Mann–Whitney U-test; categorical variables were presented as counts (percentages) and compared using the Chi-square test (χ² test). All P-values were based on two-sided tests, and P < 0.05 was considered to indicate statistical significance.

For model evaluation, a confusion matrix was first constructed, after which model performance was assessed using the area under the curve (AUC) of the receiver operating curves (ROC). To ensure the robustness of the evaluation on the test set, the 95% confidence intervals (CIs) for the AUCs were calculated using the bootstrapping method with 1,000 resampling iterations. Furthermore, to rigorously evaluate the statistical significance of performance improvements, the DeLong test was applied and the differences in AUCs between the constructed unimodal and multimodal prediction models was calculated. Moreover, to comprehensively examine the overall capability of the models in the dual-task scenario, we calculated the Joint AUC (C-index) for co-stratifying both irAE and treatment response. Pairwise comparisons of the Joint AUCs against the optimal model were conducted using the DeLong test. Additional evaluation metrics included accuracy, recall, specificity, positive predictive value (PPV), and negative predictive value (NPV). To quantify the contribution of clinical and CT features to model predictions, SHapley Additive exPlanations (SHAP) analysis was performed [35].

The specific formulas used was as follows:


$$\begin{array}{c} \begin{array}{c} \text{Accuracy}=\frac{\text{TP}+\text{TN}}{\text{TP}+\text{TN}+\text{FP}+\text{FN}} \end{array} \end{array}$$ (2)

$$\begin{array}{c} \begin{array}{c} \text{Recall}=\frac{\text{TP}}{\text{TP}+\text{FN}} \end{array} \end{array}$$ (3)

$$\begin{array}{c} \begin{array}{c} \text{Specificity}=\frac{\text{TN}}{\text{TN}+\text{FP}} \end{array} \end{array}$$ (4)

$$\begin{array}{c} \begin{array}{c} \text{Precision}=\frac{\text{TP}}{\text{TP}+\text{FP}} \end{array} \end{array}$$ (5)

$$\begin{array}{c} \begin{array}{c} \text{F1}-\text{Score}=\text{2}*\frac{\text{Precision}*\text{Recall}}{\text{Precision}+\text{Recall}} \end{array} \end{array}$$ (6)

Where, TP represents true positives, TN represents true negatives, FP represents false positives, and FN represents false negatives.

## 3 Results

### 3.1 Cohort clinical baseline

A total of 333 patients with advanced NSCLC who received PD-1 inhibitor treatment during June 2018–2022 were enrolled. The patients were randomly assigned to the training (n = 266) and test (n = 67) sets at an 8:2 ratio. The results are summarized in **Table 1**.

**Table 1:** Baseline characteristics of patients in the datasets.

	All patients (N = 333)	Cohort 1 (Training set) (n = 266)	Cohort 2 (Testing set) (n = 67)	*P value*
Age (years)				
≤63	171 (51.4%)	135 (50.8%)	36 (53.7%)	0.765
>63	162 (48.6%)	131 (49.2%)	31 (46.3%)	
Sex				
Female	79 (23.7%)	67 (25.2%)	12 (17.9%)	0.275
Male	254 (76.3%)	199 (74.8%)	55 (82.1%)	
Smoking status				
Never	142 (42.6%)	112 (42.1%)	30 (44.8%)	0.797
Current/Former	191 (57.4%)	154 (57.9%)	37 (55.2%)	
ECOG value				
0	64 (19.2)	45 (16.9)	19 (28.4)	0.051
≥1	269 (80.8)	221 (83.1)	48 (71.6)	
Histology				
Non-SCC	229 (68.8%)	175 (65.8%)	54 (80.6%)	0.029
SCC	104 (31.2%)	91 (34.2%)	13 (19.4%)	
Differentiation				
Poorly	249 (74.8%)	194 (72.9%)	55 (82.1%)	0.166
Moderately/Well	84 (25.2%)	72 (27.1%)	12 (17.9%)	
EGFR mutation				
No	282 (84.7%)	230 (86.5%)	52 (77.6%)	0.108
Yes	51 (15.3%)	36 (13.5%)	15 (22.4%)	
PD-L1 TPS				
<1%	29 (8.71%)	19 (7.14%)	10 (14.9%)	0.240
1%–50%	134 (40.2%)	108 (40.6%)	26 (38.8%)	
≥50%	106 (31.8%)	86 (32.3%)	20 (29.9%)	
Unknown	64 (19.2%)	53 (19.9%)	11 (16.4%)	
LIPI				
0	177 (53.2%)	140 (52.6%)	37 (55.2%)	0.808
1/2	156 (46.8%)	126 (47.4%)	30 (44.8%)	
PLR	160 [114, 233]	158 [113, 227]	180 [117, 254]	0.318
MLR	0.37 [0.28, 0.56]	0.37 [0.27, 0.56]	0.38 [0.32, 0.51]	0.186
SII	658 [401, 1109]	652 [407, 1063]	662 [373, 1221]	0.904
PNI	50.3 [46.0, 53.5]	50.3 [45.9, 53.6]	50.5 [46.2, 53.4]	0.713
Hb	127 [117, 139]	127 [115, 139]	129 [122, 138]	0.221
CEA	5.18 [2.96, 20.4]	5.12 [2.96, 20.8]	5.25 [2.96, 18.8]	0.847
Bone metastasis				
No	205 (61.6%)	166 (62.4%)	39 (58.2%)	0.624
Yes	128 (38.4%)	100 (37.6%)	28 (41.8%)	
Liver metastasis				
No	295 (88.6%)	242 (91.0%)	53 (79.1%)	0.012
Yes	38 (11.4%)	24 (9.0%)	14 (20.9%)	
Brain metastasis				
No	261 (78.4%)	210 (78.9%)	51 (76.1%)	0.736
Yes	72 (21.6%)	56 (21.1%)	16 (23.9%)	
Type of treatment				
Anti-PD-1 monotherapy	32 (9.61%)	24 (9.02%)	8 (11.9%)	0.607
Anti-PD-1 + chemotherapy	215 (64.6%)	174 (65.4%)	41 (61.2%)	
Anti-PD-1 + anti-angiogenesis	23 (6.91%)	20 (7.52%)	3 (4.48%)	
Anti-PD-1 + chemotherapy + antiangiogenesis	63 (18.9%)	48 (18.0%)	15 (22.4%)	
Treatment response				
Non-responder	217 (65.2%)	170 (63.9%)	47 (70.1%)	0.415
Responder	116 (34.8%)	96 (36.1%)	20 (29.9%)	
Treatment line				
First-line	152 (45.6%)	114 (42.9%)	38 (56.7%)	0.058
Second/Third-line	181 (54.4%)	152 (57.1%)	29 (43.3%)	
No. of irAEs				
None	146 (43.8%)	118 (44.4%)	28 (41.8%)	0.809
Grade ≥3	187 (56.2%)	148 (55.6%)	39 (58.2%)	

P-value for comparisons between the training set and internal validation set; SCC, Squamous Cell Carcinoma; ECOG, Eastern Cooperative Oncology Group; LIPI, Lung Immune Prognostic Index; PLR, Platelet-to-Lymphocyte Ratio; MLR, Monocyte-to-Lymphocyte Ratio; SII, Systemic Immune-Inflammation Index; PNI, Prognostic Nutritional Index; Hb, Hemoglobin; CEA, Carcinoembryonic Antigen

Of all, 48.6% (n = 162) patients were of age > 63 years; 23.7% (n = 79) were female; 42.6% (n = 142) were never smokers; 80.8% (n = 269) had an ECOG score of ≥1; 31.2% (n = 104) had squamous cell carcinoma; 74.8% (n = 249) tumors were poorly differentiated; EGFR mutations were detected in 15.3% (n = 51) of the patients; 53.2% patients had an optimal LIPI score, and the median PNI was 50.3 [46.0, 53.5]. The bone, liver, and brain metastases incident rate of the enrolled patients were 38.4%, 11.4%, and 21.6%, respectively; 31.8% (n = 106) had PD-L1 TPS ⩾50%; only 9.61% (n = 32) received anti-PD-1 monotherapy; and 64.6% (n = 215) received anti-PD-1 combination chemotherapy. First-line treatment was administered to 45.6% (n = 152) of all patients, and 34.8% (n = 116) responded to ICI treatment.

On comparing the training and testing sets, in addition to the differences in the proportion of patients with squamous cell carcinoma (training set: n = 91; 34.2%, testing set: n = 13; 19.4%; P = 0.029) and liver metastasis (training set: n = 24; 9.0%, testing set: n = 14; 20.9%; P = 0.012), and no differences were detected in other clinical characteristics and hematological indices.

### 3.2 Evaluation of unimodal machine-learning models

**Fig 2:**
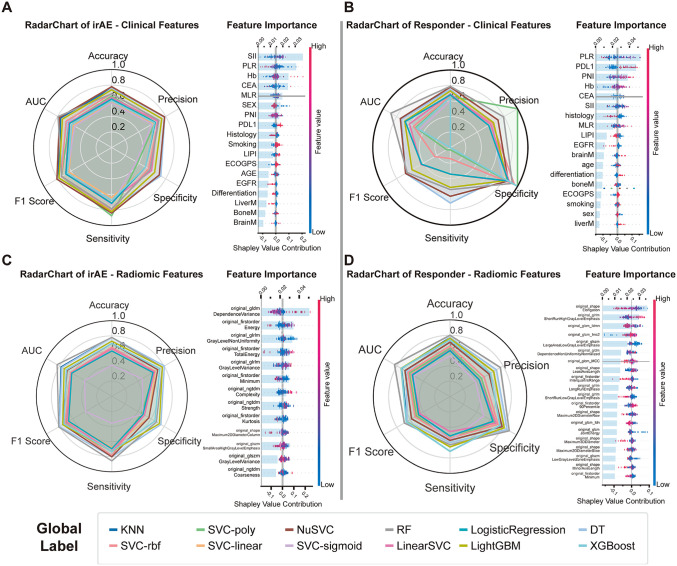
RadarChart and SHAP evaluation of unimodal machine-learning model for irAEs/ICI responder prediction. For each panel, the RadarChart (left panel) shows six diagnostic indices (i.e., accuracy, precision, specificity, sensitivity, F1 score, and AUC) in the test set. The SHAP (right panel) shows the contribution of different features in evaluating the test set. **A.** Evaluation of CML-irAE model; **B.** Evaluation of CML-responder model; **C.** Evaluation of RML-irAE model; **D.** Evaluation of RML-responder model. CML, Clinical feature-based unimodal machine-learning model; RML, Radiomics-based unimodal machine-learning model.

For the clinical and imaging treatments included in this study, two unimodal prediction models were established to predict “ICI responder” and “irAEs,” respectively. The results demonstrated that the RF models based on unimodal features exhibited good performance in predicting both ICI responder and irAEs (Fig 2A–2B). For the clinical feature-based machine-learning model (CML), AUC reached 0.793 (0.703–0.871) and 0.89 (0.813–0.942) for predicting irAE (CML-irAEs) and ICI responder (CML-responder) (**S2****A and** **S2B Fig****)**, respectively. Further analysis using SHAP revealed that the top 8 features contributing significantly to the prediction of irAEs in the clinical feature model were SII, PLR, Hb, CEA, MLR, SEX, PNI, and PD-L1. For the clinical feature model predicting ICI responder, the top 8 predictive features were PLR, PD-L1, PNI, Hb, CEA, SII, histology, and MLR **(Fig 2A–**2B).

Leveraging radiomics features to construct the radiomics feature-based machine-learning model (RML), the RF model demonstrated robust performance (**Fig 2C–**2D). Specifically, in the task of predicting irAEs (RML-irAEs), the model achieved an AUC of 0.838 (95% CI: 0.751–0.914), and when it came to identifying ICI responders (RML-responder), the AUC was 0.836 (95% CI: 0.738–0.916), with these results being detailed in **S2****C and** **S2D Fig**.

### 3.3 Performance evaluation of deep-learning models based on CT images

**Fig 3:**
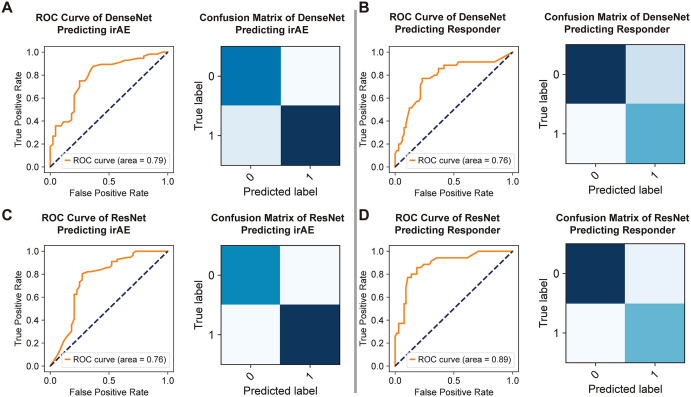
Receiver operating characteristic (ROC) curves and confusion matrix evaluation of the deep-learning model for irAEs/ICI responder prediction. **A.** Evaluation of CTDL-DenseNet-irAE model, **B.** Evaluation of CTDL-DenseNet-responder model, **C.** Evaluation of CTDL-ResNet-irAE model, **D.** Evaluation of CTDL-ResNet-responder model. CTDL, CT-based unimodal deep-learning model based on different frameworks**.**

To further explore the potential of CT image data in predicting the ICI responder and the occurrence of irAEs using pre-processed 4D CT image data, two CT-based deep-learning models (CTDL) were constructed using the DenseNet-121 and ResNet-18 framework. The prediction model based on DenseNet achieved an AUC of 0.788 (0.694–0.874) for irAEs (CTDL–DenseNet-irAEs, **Fig 3A**) prediction and an AUC of 0.755 (0.640–0.853) for ICI responder prediction (CTDL–DenseNet-responder, **Fig 3B**). Similarly, the prediction model based on ResNet achieved an AUC of 0.758 (0.661–0.861) for irAEs prediction (CTDL–ResNet-irAEs, **Fig 3C**) and an impressive AUC of 0.885 (0.814–0.954) for ICI responder prediction (CTDL–ResNet-responder, **Fig 3D**). The results indicated that the CT-based deep-learning models demonstrated a superior performance of AUC in comparison to the CML and RML models in predicting both the ICI responder status and irAEs outcomes (**Table 2**).

**Table 2:** Prediction metrics of different models for predicting irAEs and treatment response in the External Test Set.

Prediction	Model Name	Accuracy	Precision	Recall	F1-Score	AUC (95% CI)	P-value
irAE	CML-irAEs	0.69	0.687	0.677	0.678	0.793 (0.703–0.871)	0.314
	RML-irAEs	0.78	0.782	0.769	0.773	0.838 (0.751–0.914)	0.8293
	CTDL-DenseNet-irAEs	0.75	0.747	0.75	0.748	0.788 (0.694–0.874)	0.293
	CTDL-ResNet-irAEs	0.76	0.756	0.756	0.756	0.758 (0.661–0.861)	0.1443
	CRML-irAEs	0.75	0.747	0.75	0.748	0.816 (0.724–0.895)	0.5522
	CDML-ResNet-irAEs	0.72	0.717	0.711	0.713	0.831 (0.748–0.909)	0.731
	CDML–DenseNet-irAEs	0.79	0.789	0.783	0.785	**0.85 (0.773**–**0.918)**	Reference
Responder	CML-responder	0.79	0.791	0.733	0.748	0.885 (0.813–0.942)	0.7012
	RML-responder	0.80	0.788	0.76	0.77	0.836 (0.738–0.916)	0.6085
	CTDL-DenseNet-responder	0.77	0.752	0.77	0.757	0.755 (0.640–0.853)	0.0913
	CTDL-ResNet-responder	0.84	0.823	0.831	0.826	**0.885 (0.814**–**0.954)**	0.7117
	CRML-responder	0.80	0.78	0.78	0.78	0.848 (0.769–0.923)	0.7387
	CDML-ResNet-responder	0.78	0.791	0.712	0.727	0.86 (0.780–0.927)	0.9093
	CDML–DenseNet-responder	0.74	0.724	0.675	0.684	**0.866 (0.786**–**0.931)**	Reference

irAEs, Immune-related adverse events; CML, Clinical feature-based unimodal machine-learning model; RML, Radiomic features-based unimodal machine-learning model; CTDL, CT-based unimodal deep-learning model; CRML, Clinical–Radiomics-based multimodal machine-learning model; CDML, Clinical–DL-prediction-based multimodal machine-learning model; CI, confidence interval. Data in brackets are 95% CIs estimated by using the bootstrapping method with 1,000 resamples. “P-values indicate pairwise comparisons of AUCs against the CDML-DenseNet model, calculated using the DeLong test.

### 3.4 Evaluation of multimodal prediction models

To further enhance the performance of models predicting irAEs and ICI responders, two distinct multimodal machine-learning models were established by integrating clinical data with CT images: clinical–radiomics machine-learning models (CRML) and clinical–deep-learning machine-learning models (CDML).

**Fig 4:**
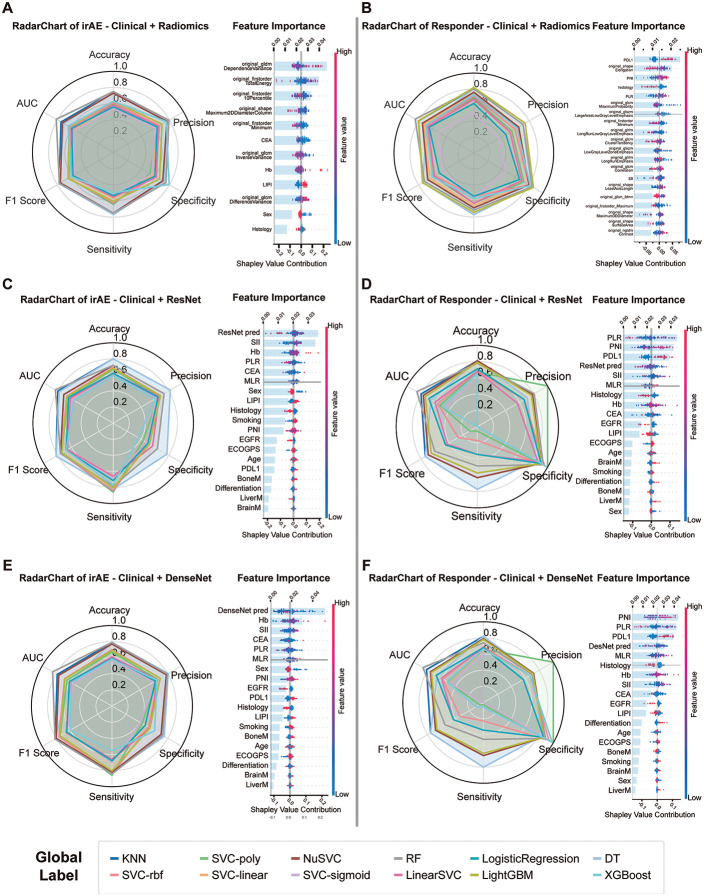
RadarChart and SHAP evaluation of the multimodal machine-learning models for irAEs/ICI responder prediction. For each panel, the RadarChart (left panel) shows six diagnostic indices (i.e., accuracy, precision, specificity, sensitivity, F1 score, and AUC) in the test set. The SHAP (right panel) depicts the contribution of different features in evaluating the test set. **A.** Evaluation of CRML-irAE model; **B.** Evaluation of CRML-responder model; **C.** Evaluation of CDML-ResNet-irAE model; **D.** Evaluation of CDML-ResNet-responder model; **E.** Evaluation of CDML–DenseNet-irAE model; **F.** Evaluation of CDML–DenseNet-responder model. CRML, Clinical–radiomics based multimodal machine-learning model; CDML, Clinical-DL-score based multimodal machine-learning model.

For the CRML models, in predicting irAEs, it reached an AUC of 0.816 (0.724–0.895) (**Fig 4A**, **S3A Fig**), which is numerically higher than the CML model (0.793; 0.703–0.871), whereas the model reached a higher performance of AUC than RML model in predicting ICI responder (CRML: 0.848 [0.769–0.923] V.S. RML: 0.836 [0.738–0.916]) (**Fig 4B**, **S3B Fig**).

For the CDML–ResNet models, the RF model revealed a moderate performance when compared to other machine-learning models (**S4****A and** **S4B Fig**), the AUC was 0.831 (0.748–0.909) and 0.86 (0.780–0.927) in predicting irAE and ICI responder, respectively (**Fig 4C-**4D). The final results of CDML–DenseNet models, which incorporated DenseNet predictions and clinical features (CDML) employed the RF framework to achieve a high level of performance (**Fig 4E-**4F). Specifically, the AUC for predicting irAE reached 0.85 (0.773–0.918), whereas the AUC for predicting ICI responder was 0.866 (0.786–0.931) (**S4****C and** **S4D Fig**), indicating the model’s predictive capabilities across different outcomes. Further analysis of the top contributing features within the optimized clinical feature+DenseNet model revealed the top 8 features for predicting irAEs: DenseNet prediction, Hb, SII, CEA, PLR, MLR, Sex, and PNI (**Fig 4E**). For the clinical feature model predicting ICI responder, the top 8 predictive features were PNI, PLR, PD-L1, DenseNet prediction, MLR, Histology, Hb, and SII (**Fig 4F**).

After analyzing all the prediction results (**Table 2**), the performance of deep-learning models was found to vary across different endpoints. For irAEs prediction, the CDML–DenseNet model achieved the best single-task performance (AUC = 0.85, 95% CI: 0.773–0.918), outperforming both standalone deep-learning models (CTDL–DenseNet: 0.788; CTDL–ResNet: 0.758) and traditional machine-learning models (CML: 0.793; RML: 0.838). However, for ICI responder prediction, the performance advantage of deep learning was inconsistent, whereas CTDL–ResNet achieved a high efficacy (AUC = 0.885, 0.814–0.954) that is comparable to the clinical feature model (CML: 0.885) and CTDL–DenseNet exhibited suboptimal performance (AUC = 0.755, 0.640–0.853), highlighting that standalone deep-learning models do not consistently surpass traditional machine-learning approaches.

Nevertheless, clinical decision-making requires simultaneous consideration of both therapeutic efficacy and toxicity risk rather than optimization of single endpoints in isolation. To address this aspect, we employed a novel joint evaluation framework using the Joint AUC metric (**Table 3**) that quantifies the model’s ability to co-stratify both irAEs and treatment response. Under this comprehensive metric, CDML–DenseNet achieved the optimal Joint AUC of 0.866 (95% CI: 0.819–0.908), which significantly outperformed standalone CTDL–DenseNet (Joint AUC: 0.787, p = 0.0126) and surpasses all unimodal models, including CML (0.847) and RML (0.854). These results demonstrate that while deep-learning alone exhibits variable performance across individual tasks, its integration with clinical features via multimodal fusion enables superior co-stratification of the dual outcomes, validating the clinical utility of our approach for balanced benefit-risk assessment.

**Table 3:** Prediction metrics of joint AUC for co-stratifying irAEs and treatment response.

Model Name	Joint AUC	95% CI	P-value
CDML–DenseNet	0.866	(0.819–0.908)	Reference
CDML-ResNet	0.866	(0.821–0.915)	Reference
RML	0.854	(0.795–0.904)	0.6023
CML	0.847	(0.791–0.894)	0.2418
CRML	0.842	(0.785–0.890)	0.3076
CTDL-ResNet	0.839	(0.781–0.892)	0.3227
CTDL-DenseNet	0.787	(0.720–0.850)	0.0126

CML, Clinical feature-based unimodal machine-learning model; RML, Radiomic features-based unimodal machine-learning model; CTDL, CT-based unimodal deep-learning model; CRML, Clinical–Radiomics-based multimodal machine-learning model; CDML, Clinical–DL-prediction-based multimodal machine-learning model; AUC, Area Under the Curve; CI, Confidence interval. Data in brackets are 95% CIs estimated by using the bootstrapping method with 1,000 resamples. P-values indicate pairwise comparisons of Joint AUCs against the CDML–DenseNet model, calculated using the DeLong test.

Considering that predicting irAE and ICI responders demand good model performance, the CDML–DenseNet model was selected as an ideal model.

### 3.5 PNI as a potential clinical indicator in predicting irAEs

**Fig 5:**
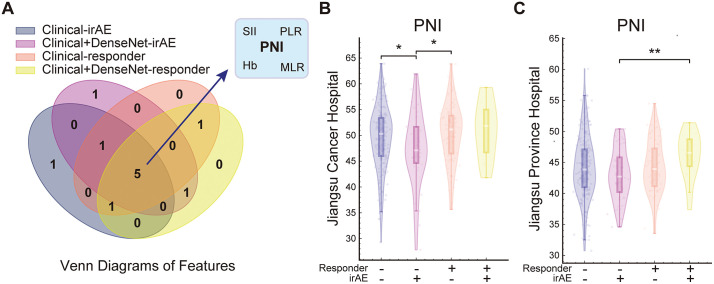
Clinical features contributing to both predicting irAE and ICI responder. **A.** Venn diagrams of the clinical features that contributed to both predicting irAEs and ICI responder; Subgroups analysis of the prognostic nutritional index (PNI) based on ICI responder and irAEs status in the cohorts of **(B)** the Jiangsu Cancer Hospital and **(C)** the First Affiliated Hospital with Nanjing Medical University.

To identify clinical features contributing to both irAE and ICI response prediction, we extracted the top eight high-contribution features from the two CML models and the two CDML–DenseNet models (**Fig 5A**). Across all high-performing models, a consistent set of variables emerged, including the nutritional index PNI and the hematologic inflammatory surrogates SII, Hb, PLR, and MLR. This concordance suggests that systemic inflammatory status and nutritional reserves are mechanistically intertwined to both therapeutic efficacy and susceptibility to irAEs.

Subgroup analyses stratified by ICI response and irAEs occurrence showed that SII, Hb, PLR, and MLR did not differ significantly among the four subgroups (**S5 Fig**). In contrast, PNI exhibited a distinct distribution. Non-responders who developed irAEs (PNI 46.8 ± 8.8) had significantly lower values than both responders without irAEs (PNI 50.3 ± 5.6, P < 0.05) and responders with irAEs (PNI 51.1 ± 5.4, P < 0.05). No significant differences were observed between these latter groups and the non-responder without irAEs group (PNI: 49.55 ± 6.04) (**Fig 5B**).

A similar pattern was observed in an external cohort from Jiangsu Province Hospital, wherein the responder without irAEs experienced a PNI of 43.06 ± 3.99, which was significantly lower than that of the responders with irAEs (PNI: 46.15 ± 3.64) (**Fig 5C**).

## 4 Discussion

### 4.1 Strengths and clinical implications

In this study, we established a cohort of patients with detailed records of ICI treatment history and irAEs events. Based on the evaluation of several unimodal and multimodal models, the CDML multimodal model, integrating clinical feature and CT-based deep-learning prediction, was selected for simultaneously predicting ICI responder and irAEs. Finally, through analysis of feature contributions, the PNI was identified as a potential clinical indicator for detecting patients unlikely to benefit from ICIs treatment and at risk of developing irAEs.

First, we established multiple models based on different medical data. Specifically, three unimodal (i.e., CML, RML, CTDL) and 2 multimodal (i.e., CRML, CDML) prediction models were established. Aligning with the advantages of deep learning in medical imaging analysis, as proposed by Dayarathna et al.[36], two CTDL models based on the deep-learning framework ResNet-18 and DenseNet-121, demonstrated significant potential in extracting imaging features [37,38]. The CTDL–ResNet model achieved an AUC of 0.885 in predicting ICI responder, which is comparable to that of the CML model (AUC = 0.885) and numerically higher than that of the RML model (AUC = 0.836).

Moreover, past studies have demonstrated that integrating multimodal data markedly enhances model performance [39]. In our research, the multimodal models, after gathering the clinical features and the CT-derived features (radiomics features/deep-learning prediction), displayed a more robust performance in both predicting ICI responder and irAEs. Our results demonstrated that the CDML–DenseNet model, based on the combination of clinical features and DenseNet prediction, achieved the numerically highest performance in predicting irAEs (AUC = 0.85). In predicting ICI responder, the CDML–DenseNet model, through multimodal fusion, by integrating cross-dimensional information, reached an AUC of 0.866, indicating the potential to overcome the limitations of single-modal and single-task predictions. These findings suggests that deep-learning models complement the local immunological information not captured by clinical features and achieve robust AUC performance in both irAEs and ICI responder prediction. Second, in clinical practice, balancing predictions of therapeutic efficacy and toxicity risk is essential for individualized treatment planning [40]. Our models were designed to predict both irAEs and ICI response simultaneously within the same dataset. This dual-task approach goes beyond simple performance comparison, leveraging the relationship between irAEs and treatment efficacy to enhance clinical utility. In our study, all multimodal architectures achieved AUCs > 0.8 for both tasks, indicating stable performance in the integrated evaluation of ICI responder and irAEs.

From the perspective of clinical translation and considering the differences in medical resources accessible to different medical centers, neglecting underserved areas and their specific context may further the digital divide [41]. The difference in the technological infrastructure between high- and low-level hospital further creates a barrier in allowing AI integration [42]. In our study, the proposed multi-level diagnostic prediction model offers differentiated solutions. Primary hospitals can rapidly screen high-risk patients based on clinical features using CML models, whereas for medical centers with CT-imaging analysis capabilities, the predictive accuracy can be enhanced by incorporating radiomic features using the unimodal RML model and the multimodal CRML model. For high-level medical centers that can deploy localized deep-learning models, clinicians can achieve more precise individualized risk stratification by using the unimodal CTDL model and the multimodal CDML models. Institutions at each level can select appropriate prediction schemes based on their own resource status, thereby achieving a risk-benefit balance in individualized ICI treatment in clinical settings.

Third, in multiple predictive models of this study, the PNI, calculated as “serum albumin (g/L)+5* absolute lymphocyte count (10⁹/L),” played a pivotal role as a core shared feature indicator. Similar observation was reported in an external cohort from Jiangsu Province Hospital, further illustrating the potential of the PNI. When compared to the conventional inflammatory markers, the PNI, which provides a comprehensive assessment of the host’s microenvironment by integrating nutritional (albumin) and immunological dimensions, demonstrates a more stable cross-modal predictive performance. It has been widely validated as an effective predictive indicator for patients undergoing immunotherapy. Li et al. reported PNI as an indicator for predicting ICI response [43]. A recent study by Xia et al. illustrated the association of pretreatment PNI with poorer prognosis, which can be applied as a biomarker to identify patients who can obtain a survival benefit from ICI therapy [44]. Dynamic monitoring of the PNI can help clinicians better evaluate the risk of irAE, potentially enhancing patient prognosis. Further analysis of PNI revealed its utility in helping clinicians identify patients who did not respond to ICIs, but experienced irAEs. However, for these patients, ICI administration should be approached with greater caution, more rigorous evaluation, and more frequent monitoring.

### 4.2 Limitations and future directions

This study, despite its potential in clinical translation, has several limitations that should be addressed in the future. First, owing to ethical approvals and technical barriers, model-level external validation of the complete CDML–DenseNet framework was not performed. Although the shared clinical feature PNI was validated as a reproducible predictor in an independent external cohort, prospective validation in larger, harmonized multi-center cohorts warrants substantiation of the generalizability of the integrated model. Second, this study only studied the characteristics of the primary lesion within the lungs, and future studies should incorporate more clinically relevant data to obtain a relatively more comprehensive assessment.

## 5 Conclusion

This study has demonstrated that the deep-learning-based CDML–DenseNet multimodal model, which integrates CT deep-learning prediction with clinical data, can effectively predict ICI responder and the risk of irAEs in NSCLC. The performance of this model, particularly within the dual-task co-stratification framework (Joint AUC = 0.866), demonstrates superior balanced discrimination compared with single-modal approaches, validating its clinical utility for simultaneous benefit-risk assessment. The PNI, serving as a common core indicator for both irAEs prediction models and ICI responder prediction models, provides clinicians with a simple and convenient basis for initial risk assessment. Utilizing routine clinical indicators, this economically feasible and convenient model aids in optimizing immunotherapy decision-making.

**Significance**

This study establishes CDML–DenseNet as the first multimodal framework capable of predicting irAEs and ICI response in parallel. Its pivotal shared predictor, PNI, empowers clinicians to flag non-responders experiencing irAEs and to refine therapeutic decisions accordingly.

## Supporting information

S1 Fig Pre-processing pipeline for CT scans. An illustration of the construction of 4D-stacked CT image tensors for deep-learning model input. **(Left panel)** Original CT images were acquired with a LightSpeed VCT scanner (matrix: 512 × 512 pixels, original slice thickness: 1.25 mm, in-plane resolution: 0.6992 × 0.6992 mm). **(Middle panel)** Images underwent resampling to an isotropic resolution of 1 × 1 × 1 mm^3^ using trilinear interpolation. Lesion regions of interest (ROIs) were manually segmented by two professional radiologists, with consensus achieved through discussion with a senior radiologist in case of discrepancies. Multi-window reconstructions were generated: **(Top panel)** Lung window (window width: 1,400 HU, window level: -500 HU) and **(Bottom panel)** Abdomen/Mediastinum window (window width: 350 HU, window level: 40 HU). **(Right panel)** Final 4D-stacked tensor construction (height × width × depth × channels) by stacking the original image, lung window, and mediastinum window sequences as separate channels. Label segmentation masks were employed to extract lesion-focused regions for subsequent analyses.
(TIF)

S2 Fig Comparison of AUC and accuracy from 12 machine-learning algorithms, and the ROC and confusion matrix for the selected models. For each panel, the top-left panel displays AUCs for the 12 algorithms and the top-right panel shows the corresponding accuracies; the bottom-left panel shows the ROC curve of the algorithm selected as the final model, the solid line indicates model performance, and the diagonal dashed line denotes random classification, with the AUC annotated; the bottom-right panel presents the confusion matrix, wherein the rows denote true labels and columns denote predicted labels, and the color intensity reflects the number of cases, with darker shading indicating higher counts. **A.** CML-irAEs; **B.** CML-responder; **C.** RML-irAEs; **D.** RML-responder. CML, Clinical feature–based unimodal machine-learning model; RML, Radiomics-based unimodal machine-learning model.
(TIF)

S3 Fig Comparison of AUC and accuracy from 12 machine-learning algorithms, and the ROC and confusion matrix for the selected models. For each panel, the top-left panel displays AUCs for the 12 algorithms and the top-right panel the corresponding accuracies; the bottom-left panel shows the ROC curve of the algorithm selected as the final model, where the solid line indicates model performance and the diagonal dashed line denotes random classification, with the AUC annotated; the bottom-right panel presents the confusion matrix, wherein the rows denote true labels and columns denote predicted labels, whereas the color intensity reflects the number of cases, with darker shading indicating higher counts. **A.** CRML-irAEs; **B.** CRML-responder. CRML, Clinical–radiomics based multimodal machine-learning model.
(TIF)

S4 Fig Comparison of AUC and accuracy from 12 machine-learning algorithms, and the ROC and confusion matrix for the selected models. For each panel, the top-left panel displays AUCs for the 12 algorithms and the top-right panel the corresponding accuracies; the bottom-left panel shows the ROC curve of the algorithm selected as the final model, where the solid line indicates model performance and the diagonal dashed line denotes random classification, with the AUC annotated; the bottom-right panel presents the confusion matrix, wherein the rows denote true labels and the columns denote predicted labels, and the color intensity reflects the number of cases, with darker shading indicating higher counts. **A.** CDML-ResNet-irAEs; **B.** CDML-ResNet-responder; **C.** CDML–DenseNet-irAEs; **D.** CDML–DenseNet-responder. CDML, Clinical–DL-score based multimodal machine-learning model.
(TIF)

S5 Fig Violin plots of hematologic and inflammation-related indices across groups stratified by responder status and irAEs status. **A.** Hemoglobin (Hb); **B.** Systemic immune inflammation Index (SII); **C.** Platelet-to-lymphocyte ratio (PLR); **D.** Monocyte-to-lymphocyte ratio (MLR).
(TIF)

S1 Table Full list of 107 radiomic features extracted from CT images. (DOCX)
