# Supplementary material for: AI-driven dual-task prediction model for co-stratifying efficacy and toxicity in NSCLC immunotherapy
Source: PLOS Digit Health. 2026 Aug 13;5(8):e0001568. doi: 10.1371/journal.pdig.0001568 (PMC13472448; doi:10.1371/journal.pdig.0001568)
Supplement: Supplementary file 1 [file pdig.0001568.s006.docx]

**Table S1** Full list of 107 radiomic features extracted from CT images

| No | Feature Class | Feature Name |
| --- | --- | --- |
| 1 | shape | Elongation |
| 2 | shape | Flatness |
| 3 | shape | LeastAxisLength |
| 4 | shape | MajorAxisLength |
| 5 | shape | Maximum2DDiameterColumn |
| 6 | shape | Maximum2DDiameterRow |
| 7 | shape | Maximum2DDiameterSlice |
| 8 | shape | Maximum3DDiameter |
| 9 | shape | MeshVolume |
| 10 | shape | MinorAxisLength |
| 11 | shape | Sphericity |
| 12 | shape | SurfaceArea |
| 13 | shape | SurfaceVolumeRatio |
| 14 | shape | VoxelVolume |
| 15 | firstorder | 10Percentile |
| 16 | firstorder | 90Percentile |
| 17 | firstorder | Energy |
| 18 | firstorder | Entropy |
| 19 | firstorder | InterquartileRange |
| 20 | firstorder | Kurtosis |
| 21 | firstorder | Maximum |
| 22 | firstorder | MeanAbsoluteDeviation |
| 23 | firstorder | Mean |
| 24 | firstorder | Median |
| 25 | firstorder | Minimum |
| 26 | firstorder | Range |
| 27 | firstorder | RobustMeanAbsoluteDeviation |
| 28 | firstorder | RootMeanSquared |
| 29 | firstorder | Skewness |
| 30 | firstorder | TotalEnergy |
| 31 | firstorder | Uniformity |
| 32 | firstorder | Variance |
| 33 | glcm | Autocorrelation |
| 34 | glcm | ClusterProminence |
| 35 | glcm | ClusterShade |
| 36 | glcm | ClusterTendency |
| 37 | glcm | Contrast |
| 38 | glcm | Correlation |
| 39 | glcm | DifferenceAverage |
| 40 | glcm | DifferenceEntropy |
| 41 | glcm | DifferenceVariance |
| 42 | glcm | Id |
| 43 | glcm | Idm |
| 44 | glcm | Idmn |
| 45 | glcm | Idn |
| 46 | glcm | Imc1 |
| 47 | glcm | Imc2 |
| 48 | glcm | InverseVariance |
| 49 | glcm | JointAverage |
| 50 | glcm | JointEnergy |
| 51 | glcm | JointEntropy |
| 52 | glcm | MCC |
| 53 | glcm | MaximumProbability |
| 54 | glcm | SumAverage |
| 55 | glcm | SumEntropy |
| 56 | glcm | SumSquares |
| 57 | gldm | DependenceEntropy |
| 58 | gldm | DependenceNonUniformity |
| 59 | gldm | DependenceNonUniformityNormalized |
| 60 | gldm | DependenceVariance |
| 61 | gldm | GrayLevelNonUniformity |
| 62 | gldm | GrayLevelVariance |
| 63 | gldm | HighGrayLevelEmphasis |
| 64 | gldm | LargeDependenceEmphasis |
| 65 | gldm | LargeDependenceHighGrayLevelEmphasis |
| 66 | gldm | LargeDependenceLowGrayLevelEmphasis |
| 67 | gldm | LowGrayLevelEmphasis |
| 68 | gldm | SmallDependenceEmphasis |
| 69 | gldm | SmallDependenceHighGrayLevelEmphasis |
| 70 | gldm | SmallDependenceLowGrayLevelEmphasis |
| 71 | glrlm | GrayLevelNonUniformity |
| 72 | glrlm | GrayLevelNonUniformityNormalized |
| 73 | glrlm | GrayLevelVariance |
| 74 | glrlm | HighGrayLevelRunEmphasis |
| 75 | glrlm | LongRunEmphasis |
| 76 | glrlm | LongRunHighGrayLevelEmphasis |
| 77 | glrlm | LongRunLowGrayLevelEmphasis |
| 78 | glrlm | LowGrayLevelRunEmphasis |
| 79 | glrlm | RunEntropy |
| 80 | glrlm | RunLengthNonUniformity |
| 81 | glrlm | RunLengthNonUniformityNormalized |
| 82 | glrlm | RunPercentage |
| 83 | glrlm | RunVariance |
| 84 | glrlm | ShortRunEmphasis |
| 85 | glrlm | ShortRunHighGrayLevelEmphasis |
| 86 | glrlm | ShortRunLowGrayLevelEmphasis |
| 87 | glszm | GrayLevelNonUniformity |
| 88 | glszm | GrayLevelNonUniformityNormalized |
| 89 | glszm | GrayLevelVariance |
| 90 | glszm | HighGrayLevelZoneEmphasis |
| 91 | glszm | LargeAreaEmphasis |
| 92 | glszm | LargeAreaHighGrayLevelEmphasis |
| 93 | glszm | LargeAreaLowGrayLevelEmphasis |
| 94 | glszm | LowGrayLevelZoneEmphasis |
| 95 | glszm | SizeZoneNonUniformity |
| 96 | glszm | SizeZoneNonUniformityNormalized |
| 97 | glszm | SmallAreaEmphasis |
| 98 | glszm | SmallAreaHighGrayLevelEmphasis |
| 99 | glszm | SmallAreaLowGrayLevelEmphasis |
| 100 | glszm | ZoneEntropy |
| 101 | glszm | ZonePercentage |
| 102 | glszm | ZoneVariance |
| 103 | ngtdm | Busyness |
| 104 | ngtdm | Coarseness |
| 105 | ngtdm | Complexity |
| 106 | ngtdm | Contrast |
| 107 | ngtdm | Strength |
